# Proteolytic Activity of DegP Is Required for the *Burkholderia* Symbiont To Persist in Its Host Bean Bug

**DOI:** 10.1128/spectrum.04330-22

**Published:** 2022-12-13

**Authors:** Bohyun Jeong, Ho Am Jang, Junbeom Lee, Ha Ram Bae, Jiyeun Kate Kim

**Affiliations:** a Department of Microbiology, Kosin University College of Medicine, Busan, South Korea; b Metabolomics Research Center for Functional Materials, Kyungsung University, Busan, South Korea; South China Agricultural University

**Keywords:** DegP, biofilm formation, symbiosis, *Burkholderia*, bean bug, stress resistance

## Abstract

Symbiosis requires the adaptation of symbiotic bacteria to the host environment. Symbiotic factors for bacterial adaptation have been studied in various experimental models, including the *Burkholderia*-bean bug symbiosis model. Previously identified symbiotic factors of *Burkholderia* symbionts of bean bugs provided insight into the host environment being stressful to the symbionts. Because DegP, which functions as both a protease and a chaperone, supports bacterial growth under various stressful conditions, we hypothesized that DegP might be a novel symbiotic factor of *Burkholderia* symbionts in the symbiotic association with bean bugs. The expression level of *degP* was highly elevated in symbiotic *Burkholderia* cells in comparison with cultured cells. When the *degP*-deficient strain competed for symbiotic association against the wild-type strain, the Δ*degP* strain showed no symbiotic competitiveness. *In vivo* monoinfection with the Δ*degP* strain revealed a lower symbiont titer in the symbiotic organ than that of the wild-type strain, indicating that the Δ*degP* strain failed to persist in the host. In *in vitro* assays, the Δ*degP* strain showed susceptibility to heat and high-salt stressors and a decreased level of biofilm formation. To further determine the role of the proteolytic activity of DegP in symbiosis, we generated missense mutant DegP^S248A^ exhibiting a defect in protease activity only. The Δ*degP* strain complemented with *degP^S248A^* showed *in vitro* characteristics similar to those of the Δ*degP* strain and failed to persist in the symbiotic organ. Together, the results of our study demonstrated that the proteolytic activity of DegP, which is involved in the stress resistance and biofilm formation of the *Burkholderia* symbiont, plays an essential role in symbiotic persistence in the host bean bug.

**IMPORTANCE** Bacterial DegP has dual functions as a protease and a chaperone and supports bacterial growth under stressful conditions. In symbioses involving bacteria, bacterial symbionts encounter various stressors and may need functional DegP for symbiotic association with the host. Using the *Burkholderia*-bean bug symbiosis model, which is a useful model for identifying bacterial symbiotic factors, we demonstrated that DegP is indeed a symbiotic factor of *Burkholderia* persistence in its host bean bug. *In vitro* experiments to understand the symbiotic mechanisms of *degP* revealed that *degP* confers resistance to heat and high-salt stresses. In addition, *degP* supports biofilm formation, which is a previously identified persistence factor of the *Burkholderia* symbiont. Furthermore, using a missense mutation in a protease catalytic site of *degP*, we specifically elucidated that the proteolytic activity of *degP* plays essential roles in stress resistance, biofilm formation, and, thus, symbiotic persistence in the host bean bug.

## INTRODUCTION

Host-microbe interactions require microbial adaption to the host environment. In harmonious symbioses, microbes successfully adapt to and persist in the host without harmful effects on the host (1). Using several symbiosis model systems, bacterial mechanisms of adaptation to host environments have been studied by characterizing symbiosis-defective bacterial mutants (2). These bacterial mutants were categorized into three groups: initiation mutants, which cannot establish an initial association with the host; accommodation mutants, which can associate with the host but cannot reach a normal symbiont population; and persistence mutants, which can reach a normal symbiont population but fail to maintain that population level (2).

The *Burkholderia*-bean bug model is a very useful symbiosis model system for understanding bacterial adaptive mechanisms at the molecular level. The bean bug Riptortus pedestris, belonging to the stinkbug family Alydidae of the insect order Hemiptera, acquires its *Betaproteobacteria* symbiont, Burkholderia insecticola, from the rhizosphere environment every generation (3, 4). *Burkholderia* cells are introduced to the bean bug orally in the early-instar stage and colonize the posterior midgut (M4) of the bean bug (5). The M4 midgut is a symbiotic organ possessing numerous crypts filled with *Burkholderia* cells, enabling the exclusive symbiotic relationship between the bean bug and *Burkholderia* (6, 7). Due to the short period of symbiotic association with the host, *Burkholderia* symbionts retain their free-living ability. Thus, they are easily cultivable and subject to mutagenesis. In the laboratory, wild-type and mutant *Burkholderia* strains are introduced to host bean bugs via drinking water (8, 9). Aposymbiotic insects are generated by not providing a bacterial inoculum solution. Symbiotic insects have been compared with aposymbiotic insects, revealing the beneficial effects of the *Burkholderia* symbiont on host growth, fitness, egg production, immunity, as well as pesticide resistance (10–13). Symbiotic insects with mutant *Burkholderia* have also been compared with symbiotic insects with wild-type *Burkholderia* to investigate the effects of mutant *Burkholderia* on the host (14–18).

To understand the adaptive mechanisms of the *Burkholderia* symbiont, various mutant strains defective in different pathways, such as flagella, lipopolysaccharide (LPS), biofilm, purine biosynthesis, and polyhydroxyalkanoate (PHA) biosynthesis, have been examined (15–22). Based on the three mutant categories identified previously by Ruby (2), flagellar mutants can be classified as initiation mutants, whereas purine biosynthesis mutants are accommodation mutants, and PHA- and biofilm-defective mutants are persistence mutants (21). Interestingly, both PHAs and biofilms are bacterial mechanisms of adaptation to harsh environmental conditions. PHA granules accumulate in the cytoplasm as carbon energy storage when environmental conditions are not optimal (23). Biofilm formation is induced as a survival mechanism in many different environments, including chronic infections, protecting bacteria from harsh conditions (24–26). The fact that PHAs and biofilms are symbiotic factors of the *Burkholderia* symbiont suggests that the symbiotic condition of the host midgut is stressful to the symbionts. It is conceivable that symbiont cells must endure various stresses present in the M4 midgut, where they are densely packed and survive over 2 months of the host’s lifetime. Based on this understanding, we speculated that bacterial factors involved in stress resistance could be symbiotic factors of the *Burkholderia* symbiont providing host adaptation mechanisms.

Stressful conditions affect the protein folding of cells, generating misfolded or mislocalized proteins. All organisms prevent the dysregulation of protein folding by deploying chaperones and proteases, and these two functions are critical for bacterial survival under stressful conditions (27, 28). Widely conserved serine proteases called high-temperature requirement A (HtrA) family proteins have dual chaperone and protease functions and are involved in protein quality control under stressful conditions (29, 30). DegP is a bacterial ortholog of the HtrA family that is present in the periplasmic space of Gram-negative bacteria. DegP is composed of a protease domain and two PDZ domains. Structural studies of the mature DegP of Escherichia coli revealed that the protease catalytic site is made up of a triad of His105, Asp135, and Ser210 (31). Three monomeric DegPs are tightly associated via protease domains, forming a basic functional unit. By adopting different orientations of PDZ domains, stable hexameric DegP can be transformed to substrate-engaged 12-mer or 24-mer multimers with increased proteolytic activity (32, 33). DegP is a key protease and chaperone in extracytoplasmic protein homeostasis networks (34). Its protective roles against various stresses such as heat, oxidative, and osmotic stresses have been reported previously (35–39). In this study, we hypothesized that DegP would be critical for the *Burkholderia* symbiont to adapt to the stressful environment of the bean bug M4 midgut. As the first step to prove this hypothesis, the gene expression levels of *degP* in cultured and symbiotic *Burkholderia* cells were compared. Using a deletion mutant strain of *degP*, the functions of *degP* in *in vitro* stress resistance and *in vivo* symbiotic association were examined. Furthermore, protease-defective DegP was generated by a missense mutation and used to investigate the effects of the protease activity of DegP on stress resistance, biofilm formation, and symbiont persistence in the host.

## RESULTS

### The expression of *degP* is elevated in symbiotic *Burkholderia*, and the *degP-*deficient mutant strain has no symbiotic competitiveness against the wild-type strain.

**FIG 1:**
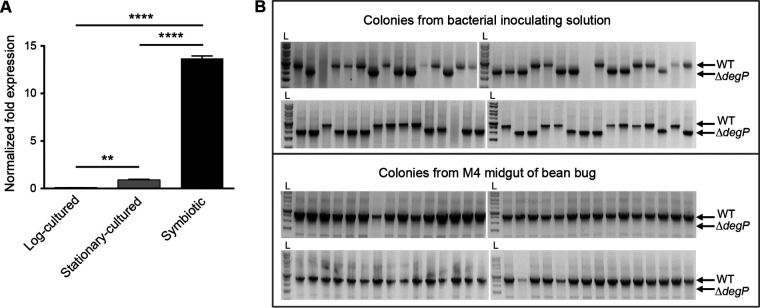
Expression of *degP* in *Burkholderia* cells and *in vivo* competition assay between wild-type and *degP* mutant strains. (A) The expression levels of *degP* were compared among cultured cells in log and stationary phases and symbiotic cells isolated from M4 midguts. Means and standard deviations (SDs) (*n* = 3) are shown as columns and error bars, respectively. Asterisks indicate statistically significant differences (**, *P < *0.01; ****, *P < *0.0001 [by one-way ANOVA with Tukey’s *post hoc* test]). (B) The symbiotic competitiveness of the Δ*degP* strain was compared with that of the wild-type (WT) strain by inoculating bean bugs with a 1:1 bacterial solution (top) followed by the determination of the proportions of the strains in M4 midguts at the 5th-instar stage (bottom). Sixty randomly selected colonies from the inoculating solution and the M4 midgut homogenate were examined for the deletion of *degP* by PCR. PCR bands of 2,499 bp and 1,884 bp indicate the wild-type and Δ*degP* strains, respectively. The data shown here are representative of results from three independent experiments. L, DNA ladder.

To address the possibility that *degP* is a symbiotic factor, we initially examined the expression levels of the *degP* gene in symbiotic *Burkholderia* cells isolated from M4 midguts in comparison to those in cultured cells in the exponential phase and stationary phase. While B. insecticola has two *degP* paralogs (BRPE64_ACDS05040 and BRPE64_ACDS19710), only one *degP* gene (BRPE64_ACDS05040) was highly expressed in symbiotic cells. Hence, the *degP* gene in this study refers to BRPE64_ACDS05040. The expression level of *degP* was elevated 15-fold in symbiotic cells relative to stationary-phase cultured cells, suggesting that *degP* could be a candidate symbiotic factor (Fig. 1A). Therefore, we generated an in-frame deletion mutant strain of *degP* to investigate the role of *degP* in the *Burkholderia*-bean bug symbiosis.

Using an *in vivo* competition assay, the symbiotic competitiveness of the Δ*degP* strain was compared with that of the wild-type strain. A 1:1 ratio (wild-type/Δ*degP* strain) bacterial solution was used to inoculate 2nd-instar bean bugs, and the ratio of wild-type to mutant cells present in the M4 midguts was analyzed at the 5th-instar stage. In three independent experiments, randomly selected colonies of *Burkholderia* symbionts from M4 midguts were all of the wild type (Fig. 1B). These results indicate that *degP* grants *B. insecticola* competitiveness in establishing a symbiotic association with bean bugs.

### *Burkholderia degP* is involved in resistance to high-temperature and high-salt stresses and biofilm formation.

**FIG 2:**
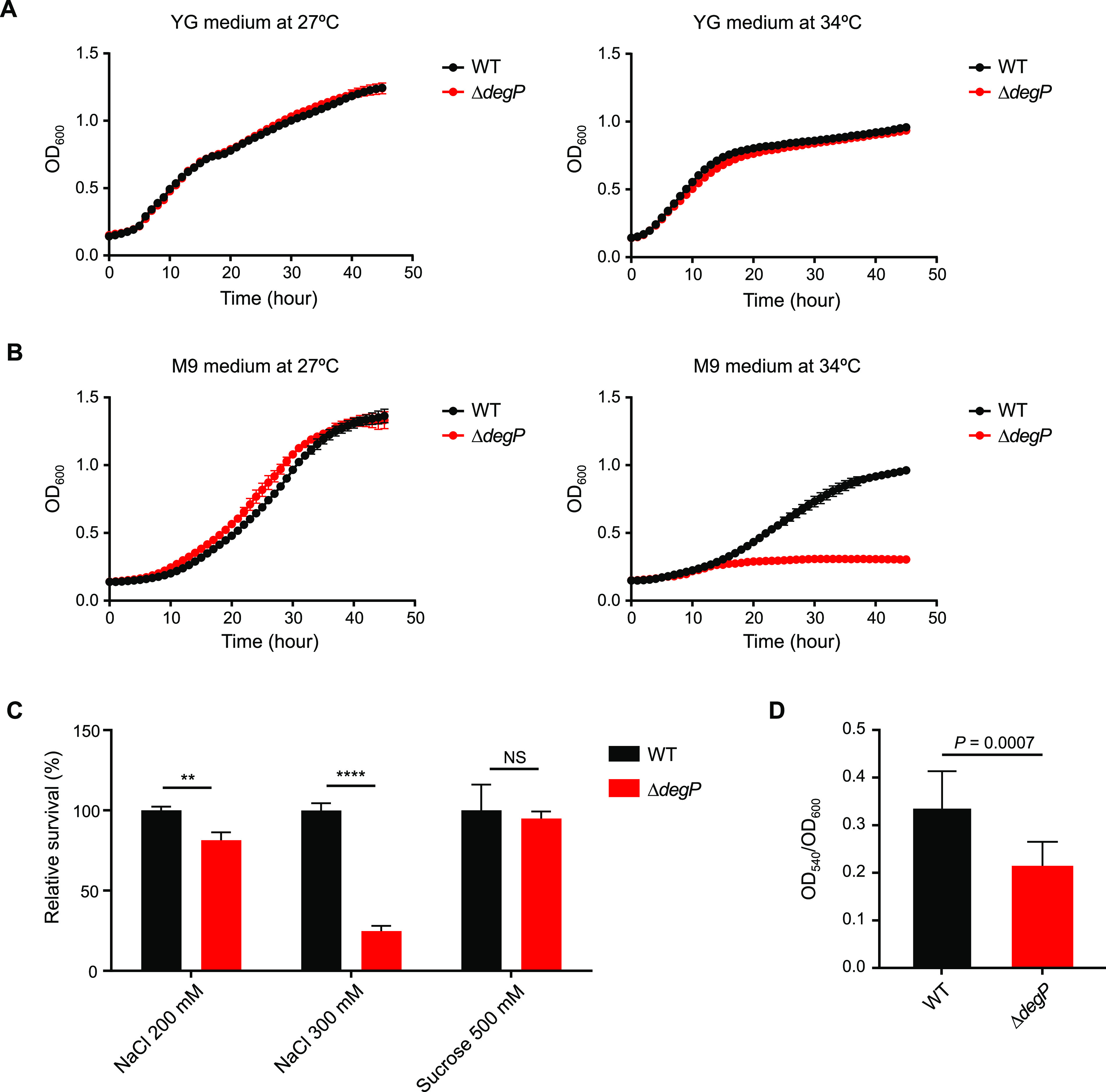
Growth, stress resistance, and biofilm formation of the wild-type and Δ*degP* strains. (A) The growth curves of the wild-type and Δ*degP* strains were similar at 27°C (left) and 34°C (right) when cultured in nutrient-rich YG medium. (B) In nutrient-poor M9 medium, the Δ*degP* strain showed a growth curve similar to that of the wild type at 27°C (left) but failed to grow at 34°C (right). Means and SDs (*n* = 3) are shown as dots and error bars, respectively. (C) The relative survival rates of the Δ*degP* strain were compared with those of the wild type using a CFU assay under high-salt conditions (200 mM and 300 mM NaCl) and high-sucrose conditions (500 mM sucrose). Means and SDs (*n* = 3) are shown as columns and error bars, respectively. Asterisks indicate statistically significant differences (**, *P < *0.01; ****, *P < *0.0001; NS, not significant [by an unpaired *t* test]). (D) Biofilm formation was quantified by measuring the OD_540_ of dissolved crystal violet divided by the bacterial OD_600_. Means and SDs (*n* = 10) are shown as columns and error bars, respectively. The *P* value from an unpaired *t* test is shown on the graph.

To understand the symbiotic functions of *degP*, we assessed the *in vitro* characteristics of the Δ*degP* deletion mutant strain. Because DegP has a well-established high-temperature requirement (hence the name HtrA), the *B. insecticola* Δ*degP* strain was tested for its growth phenotype at the optimal temperature (27°C) and a high temperature (34°C). In nutrient-rich yeast extract-glucose (YG) medium, the Δ*degP* strain displayed a normal growth pattern at both temperatures, similar to that of the wild-type strain (Fig. 2A). In nutrient-minimal M9 medium at 27°C, the Δ*degP* strain exhibited no auxotrophic phenotype, showing a growth curve similar to that of the wild-type strain. However, the Δ*degP* strain failed to grow at 34°C, indicating that *degP* is required for bacterial growth at high temperatures in minimal medium (Fig. 2B). To address the stress resistance functions of *degP*, we tested symbiosis-relevant stressors that *B. insecticola* may face during symbiotic association with the bean bug, such as surfactant and oxidative stresses and osmotic pressure. Bacterial motility and biofilm formation were also examined since they are important symbiotic factors of *B. insecticola*. The Δ*degP* strain showed susceptibilities to surfactant (sodium dodecyl sulfate) and oxidative (hydrogen peroxide) stresses similar to those of the wild-type strain (see Fig. S1 in the supplemental material). The Δ*degP* strain also exhibited motility on soft agar plates similar to that of the wild-type strain (Fig. S2). To test the resistance of *degP* to osmotic pressure, high concentrations of salt and sucrose were used. The Δ*degP* strain was susceptible to high-salt stress but not high-sucrose stress (Fig. 2C). The Δ*degP* strain exhibited a significantly lower level of biofilm formation than the wild-type strain (Fig. 2D). Biofilm formation was previously reported to be critical for *B. insecticola* to persist in the M4 midgut (16). Based on the *in vitro* characteristics of the Δ*degP* strain, we surmised that *degP* may play a role in symbiont persistence in the host midgut.

### A missense mutation generating the DegP^S248A^ protein causes a defect in only the proteolytic activity of DegP.

DegP has a chaperone function and a protease function. We further questioned which function of *Burkholderia* DegP is more critical for symbiosis with the bean bug. *B. insecticola* DegP is a 502-amino-acid (aa) protein (477-aa mature protein without a signal peptide) that has a protease catalytic site composed of His148, Asp175, and Ser248. To distinguish the protease function from the chaperone function, we generated protease-defective DegP, DegP^S248A^, by introducing a missense mutation changing serine (TCG) to alanine (GCG).

**FIG 3:**
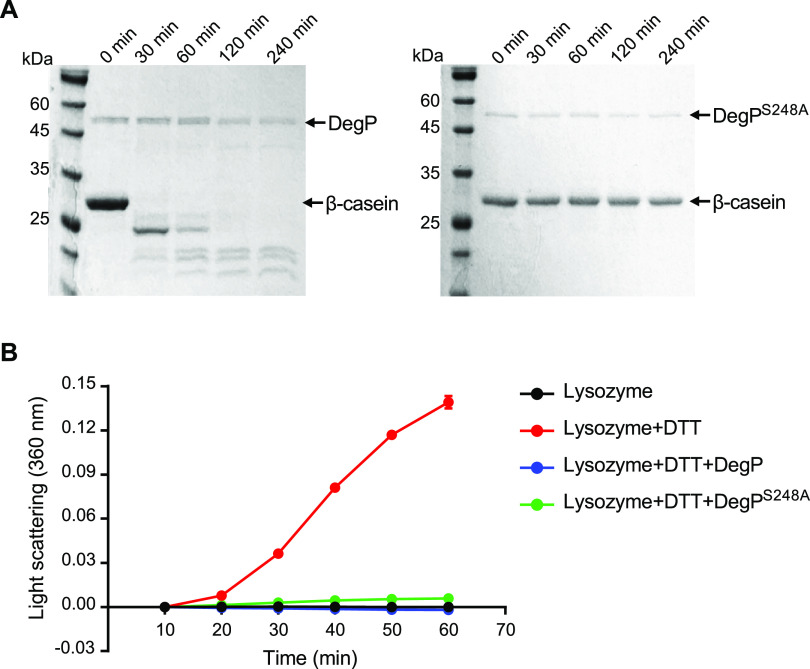
Analysis of protease and chaperone activities of wild-type DegP and protease-defective DegP^S248A^. (A) The proteolytic activities of DegP and DegP^S248A^ were measured using β-casein as a substrate. (Left) The degradation of β-casein by DegP was observed at the indicated time points using SDS-PAGE analysis. (Right) However, no degradation of β-casein was detected with DegP^S248A^. (B) The chaperone activities of DegP and DegP^S248A^ were analyzed by a light-scattering assay to detect protein aggregation. The aggregation of lysozyme denatured by DTT increased the light absorption at 360 nm in a time-dependent manner (red line). Protein aggregation was suppressed by incubation with DegP (blue line) and DegP^S248A^ (green line).

The protease activities of DegP and DegP^S248A^ were assessed by observing the degradation of β-casein. Wild-type DegP exhibited proteolytic activity against β-casein, but mutant DegP^S248A^ completely lost the proteolytic activity (Fig. 3A). However, both DegP and DegP^S248A^ exhibited similar chaperone activities, suppressing the aggregation of denatured lysozyme (Fig. 3B). These results indicate that the S248A point mutation generates DegP with a defect in proteolytic activity only and thus can be used for further experiments to determine the specific role of the proteolytic activity of DegP in the symbiotic association with the host insect.

### The *in vitro* functions of *degP* in stress resistance and biofilm formation are due to its proteolytic activity.

**FIG 4:**
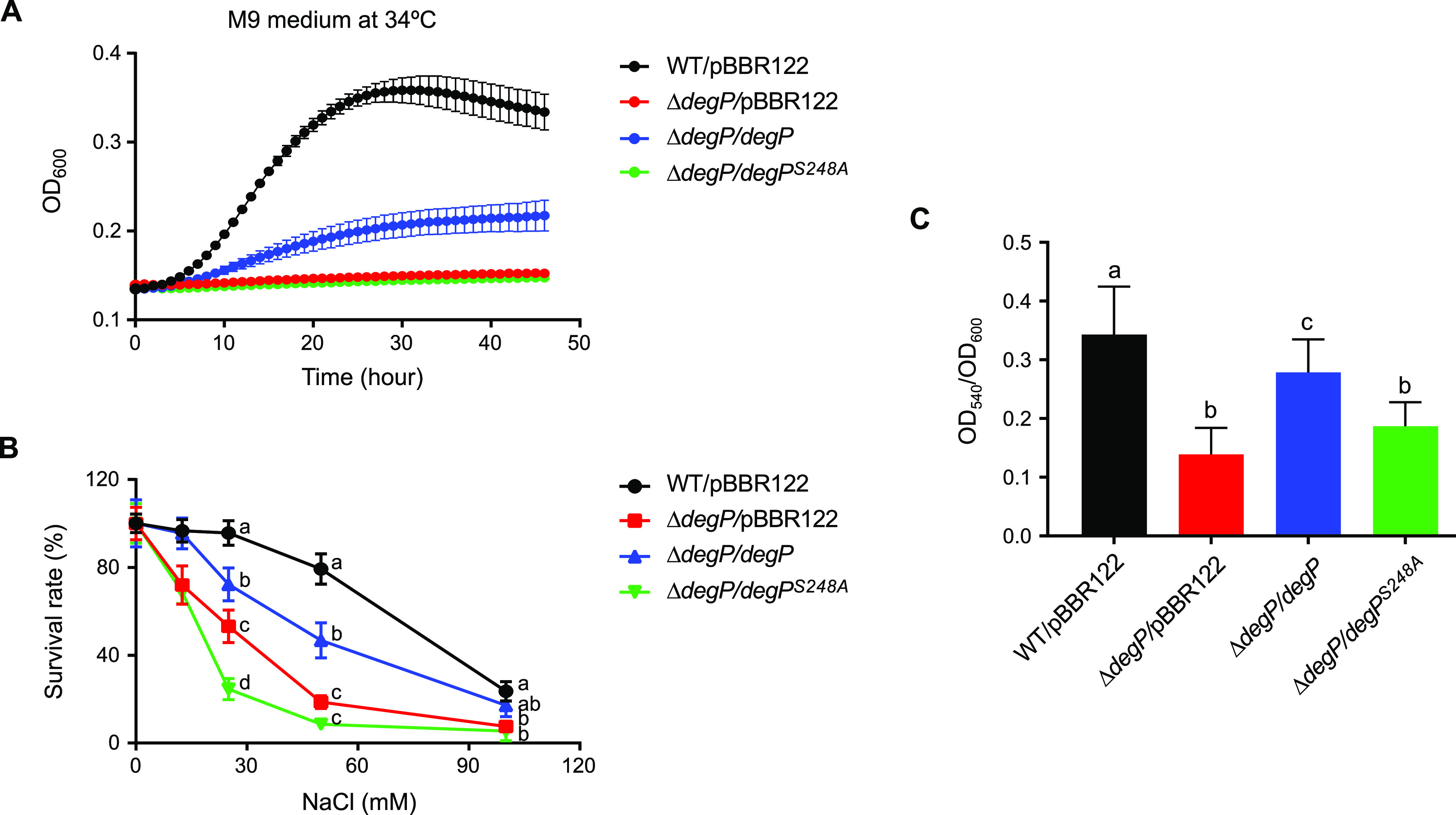
Effects of the proteolytic activity of DegP on growth at high temperatures, resistance to salt stress, and biofilm formation. (A) The bacterial growth curves in M9 medium at 34°C were compared among a wild-type strain transformed with an empty vector (WT/pBBR122) and Δ*degP* strains transformed with an empty vector (Δ*degP*/pBBR122), a vector containing a functional *degP* gene (Δ*degP*/*degP*), and a vector containing protease-defective *degP* (Δ*degP*/*degP^S248A^*). Means and standard errors of the means (*n* = 8) are shown as dots and error bars, respectively. (B) Survival rates under salt stress were analyzed by measuring the OD_600_ after culturing cells in M9 medium containing different concentrations of NaCl and comparing them with the control OD_600_ of the no-salt treatment, (OD_600_)_salt treated_/(OD_600_)_control_ (percent). Means and SDs (*n* = 3) are shown as dots and error bars, respectively. Different letters (a, b, c, and d) next to the dots indicate statistically significant differences at that NaCl concentration (*P < *0.05 [by two-way ANOVA with Tukey’s *post hoc* test]). (C) Biofilm formation was quantified by measuring the OD_540_ of dissolved crystal violet divided by the bacterial OD_600_. Means and SDs (*n* = 12) are shown as columns and error bars, respectively. Different letters (a, b, and c) on the top of the columns indicate statistically significant differences (*P < *0.05 [by one-way ANOVA with Tukey’s *post hoc* test]).

Four *B. insecticola* strains were generated to assess the role of the proteolytic activity of DegP: a wild-type strain with an empty vector (WT/pBBR122), a Δ*degP* strain with an empty vector (Δ*degP*/pBBR122), a Δ*degP* strain with a vector possessing the wild-type *degP* gene (Δ*degP*/*degP*), and a Δ*degP* strain with a vector possessing the mutant *degP^S248A^* gene (Δ*degP*/*degP^S248A^*). When we examined these strains for stress resistance and biofilm formation identified as the *in vitro* characteristics of *Burkholderia degP* (Fig. 2), the loss of the proteolytic activity of the Δ*degP*/*degP^S248A^* strain abolished the functions restored by complementation with *degP* shown in the Δ*degP*/*degP* strain, resembling the phenotypes of the Δ*degP*/pBBR122 strain (Fig. 4). The Δ*degP*/*degP* complemented strain demonstrated somewhat recovered bacterial growth at high temperatures, but the Δ*degP*/*degP^S248A^* strain could not grow at all like the Δ*degP*/pBBR122 strain (Fig. 4A). In salt stress assays, complementation with the *degP* gene restored some stress resistance, showing lower susceptibility in the Δ*degP*/*degP* strain than in the Δ*degP*/pBBR122 strain. The protease-defective Δ*degP*/*degP^S248A^* strain was most susceptible to NaCl in a dose-dependent manner, followed by the Δ*degP*/pBBR122 strain (Fig. 4B). The level of biofilm formation of the Δ*degP*/*degP^S248A^* strain was similar to that of the Δ*degP*/pBBR122 strain, being significantly lower than those of the WT/pBBR122 and Δ*degP*/*degP* strains (Fig. 4C).

### The proteolytic activity of *degP* helps *Burkholderia* symbionts persist in the M4 midgut.

**FIG 5:**
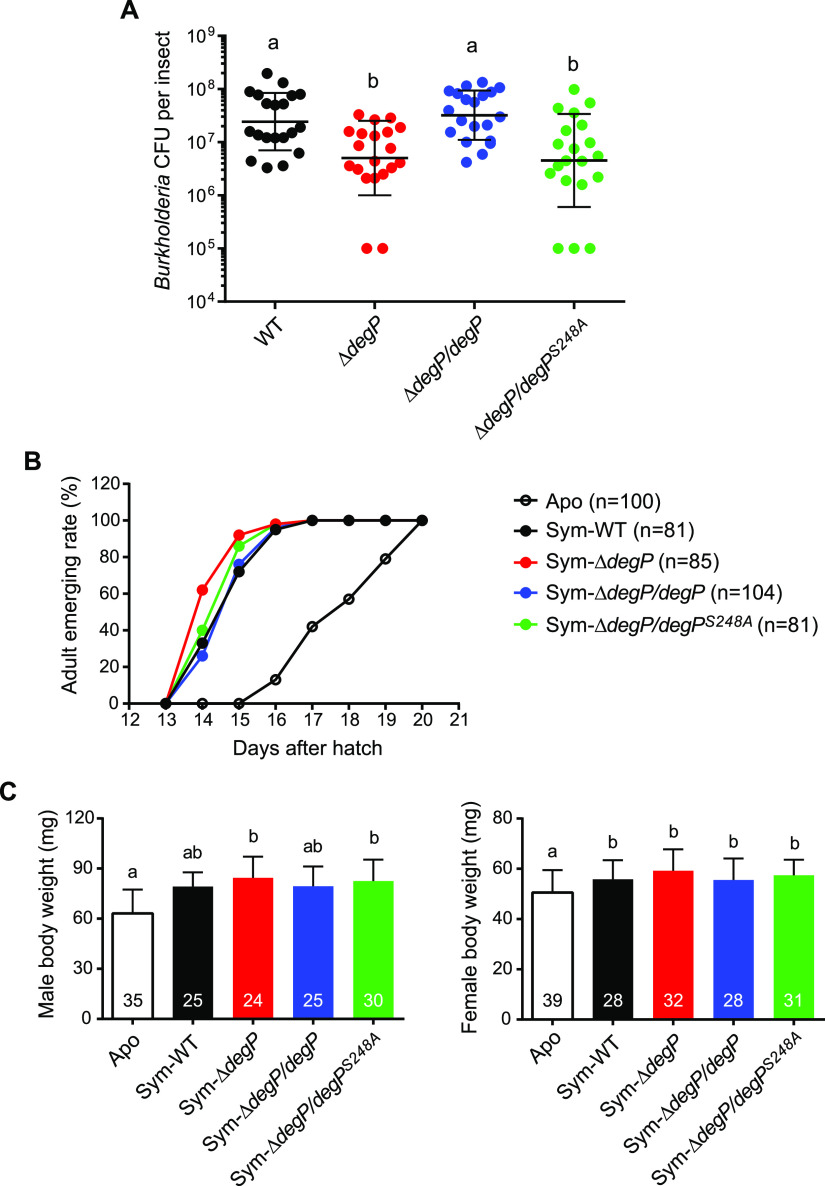
Symbiont titers of *Burkholderia* strains and their effects on host fitness. (A) Symbiont titers in 5th-instar nymphs were measured by culturing M4 midgut homogenates and counting the CFU of the *Burkholderia* symbiont. Each data point represents *Burkholderia* cell numbers per insect. Means and SDs are shown as horizontal lines and error bars, respectively. (B) The effect of each *Burkholderia* strain on host growth was analyzed by measuring adult emergence rates of symbiotic insects with the wild-type strain (Sym-WT), the Δ*degP* strain (Sym-Δ*degP*), the strain complemented with functional *degP* (Sym-Δ*degP*/*degP*), and the strain complemented with protease-defective *degP* (Sym-Δ*degP*/*degP^S248A^*) and aposymbiotic insects without *Burkholderia* symbionts (Apo). (C) The dry weights of early-adult insects (left, male; right, female) were measured as a representative fitness parameter of host bean bugs. Means and SDs are shown as columns and error bars, respectively, and sample sizes are indicated on the columns. Different letters (a and b) indicate statistically significant differences (*P < *0.05 [by one-way ANOVA with Tukey’s *post hoc* test]).

To determine how the *in vitro* functions of *degP* in stress resistance and biofilm formation affect the *in vivo* symbiotic association with the host bean bug, we inoculated bean bugs with a single strain of *B. insecticola* and compared the symbiont titers in 5th-instar M4 midguts. The symbiont titer of the Δ*degP* strain was 4-fold lower than that of the wild-type strain, indicating that the Δ*degP* strain fails to adapt to and persist in the host M4 midgut (Fig. 5A, WT and Δ*degP*). While complementation with *degP* restored the symbiont titer of the Δ*degP* strain (Fig. 5A, Δ*degP*/*degP*), complementation with protease-defective *degP* could not restore the symbiont titer, showing similar symbiont populations between the Δ*degP* and Δ*degP*/*degP^S248A^* strains (Fig. 5A, Δ*degP*/*degP^S248A^*). Even though the Δ*degP* and Δ*degP*/*degP^S248A^* strains exhibited smaller symbiont populations than those of the wild-type and Δ*degP*/*degP* strains, there were no differences in host fitness. The growth rates and body weights of bean bugs infected with the wild-type, Δ*degP*, Δ*degP*/*degP*, and Δ*degP*/*degP^S248A^* strains did not significantly differ from one another (Fig. 5B and C). These results indicate that the proteolytic activity of *degP* is essential for *B. insecticola* to persist in the host M4 midgut.

## DISCUSSION

We have demonstrated that *Burkholderia degP* is a symbiotic factor that plays an adaptive role in the association with bean bugs. The expression level of *degP* was greatly elevated in symbiotic *Burkholderia* compared to cultured *Burkholderia* cells. The deletion mutant strain of *degP* lost symbiotic competitiveness in the association with the host when in competition with the wild-type strain. In an attempt to understand the host adaptation mechanisms of *degP*, we found that the deletion of *degP* reduced resistance to salt stress and bacterial biofilm formation. *In vivo* monoinfection by the Δ*degP* strain revealed a smaller symbiont population in the M4 midgut than that of the wild-type strain. These results suggest that the Δ*degP* strain failed to persist in the M4 midgut due to reduced stress resistance and biofilm formation. Furthermore, using a missense mutation in a protease catalytic site of *degP*, we demonstrated that the proteolytic activity of *degP* plays an essential role in stress resistance, biofilm formation, and symbiotic persistence in the M4 midgut.

In Gram-negative bacteria, DegP is localized in the periplasmic space and provides protein homeostasis by inducing the proper folding, localization, and recycling of proteins through its protease and chaperone activities (29). DegP’s protease activity degrades misfolded or damaged proteins (32), and its chaperone activity is involved in sequestering and trafficking outer membrane and secreted proteins (40, 41). These activities are critical for protecting bacterial function under stressful conditions. Escherichia coli *degP* is required for growth under high-temperature (35, 36) and oxidative stress (38) conditions. In other bacteria, osmotic stress inhibited the growth of *degP*-deficient strains (37, 39). In this study, *B. insecticola* showed a requirement for *degP* for growth at high temperatures in minimal medium. The *B. insecticola* Δ*degP* strain was susceptible to osmotic pressure from high salt concentrations. On the other hand, the *B. insecticola* Δ*degP* strain was not susceptible to high sucrose concentrations and surfactant and oxidative stresses. The discrepancy of the Δ*degP* strain being susceptible to high-salt stress but not high-sucrose stress is probably due to the different effects that they have on the cells: a salt solution can induce both osmotic pressure and ionic toxicity, while a sucrose solution causes only osmotic pressure. When we consider these stress-resistant functions of *degP* in light of the symbiotic association with the bean bug, two possible outcomes could be speculated: (i) since the M4 midgut of the bean bug is regarded as being nutrient poor, *degP* may be critical for the survival of the *Burkholderia* symbiont, hence affecting bean bug fitness, at high temperatures (over 34°C), and (ii) if a high concentration of salt is present in the bean bug midguts, *degP* of *B. insecticola* may contribute to symbiont survival in the host midguts.

Biofilm formation is often critical for bacterial survival in harsh environments. Bacteria envelop their community with an extracellular polymeric matrix to protect themselves from harmful conditions. Thus, biofilm formation plays a role in the colonization of hosts by pathogenic and symbiotic bacteria. The biofilm matrix is composed of various molecules, including polysaccharides, proteins, nucleic acids, and outer membrane vesicles (OMVs). The effects of *degP* on biofilm formation seem different among bacterial species. The deletion of *degP* induced OMV production and, hence, biofilm formation in E. coli and Salmonella (42). The inactivation of Pseudomonas aeruginosa *mucD* (a *degP* homolog) caused alginate overproduction and increased OMV production (43, 44). On the contrary, mutation of *degP* affects the OMV protein composition and reduces biofilm formation in Vibrio cholerae (45). A Vibrio fischeri *degP* mutant also showed reduced OMV production and biofilm formation (46). In the Gram-positive bacteria Streptococcus mutans and Listeria monocytogenes, *degP* is required for efficient biofilm formation (47, 48). In the case of *B. insecticola*, the deletion of *degP* reduced biofilm formation, showing the role of *degP* in promoting biofilm formation.

In our previous study, we identified a gene involved in purine biosynthesis and biofilm formation, *purT*. The *Burkholderia purT* mutant strain exhibited a low level of cyclic di-GMP and, thus, a defect in biofilm formation (16). In contrast to the Δ*degP* strain, the Δ*purT* strain showed negative effects on host insects. Bean bugs infected with the Δ*purT* strain exhibited lower growth rates and lower body weights than bean bugs with wild-type symbionts. This *in vivo* dissimilarity between the Δ*degP* and Δ*purT* strains might be due to the different degrees of defects in biofilm formation. While the level of biofilm formation of the Δ*degP* strain was about 2-fold lower than that of the wild-type strain, The Δ*purT* strain showed about 13-fold-lower levels of biofilm formation than the wild-type strain. It is likely that the degrees of defects in biofilm formation affect symbiont survival in the host differently. It is also possible that the deletion of *purT* in the purine biosynthesis pathway may impair bacteria in broader aspects than the deletion of the periplasmic serine protease gene *degP*. Therefore, even though the Δ*degP* strain exhibited a decreased level of symbiont persistence in the M4 midgut, the effect of the *degP* deletion on host biology was probably not as severe as that of the *purT* deletion.

The roles of *degP* in interactions with the host have been studied more in pathogenesis than in symbiosis. In bacterium-plant symbioses, *degP* was not required for the nitrogen-fixing root nodule symbionts Bradyrhizobium japonicum and Rhizobium meliloti to establish the symbiotic association (49, 50). DegP of Vibrio fischeri, a symbiont of the Hawaiian bobtail squid, showed slightly defective colonization compared to wild-type cells (46). The obligate endosymbiont *Buchnera* of some aphids such as Acyrthosiphon pisum and Schizaphis graminum possesses the *degP* gene in its reduced genome, and *Buchnera* in another aphid, Cinara cedri, lost the *degP* gene in its genome (51). The role of DegP in bacterial pathogenesis has been reported for various pathogens. In Streptococcus pyogenes, DegP plays a role in processing the virulence factors SpeB and streptolysin S (52). In Bordetella pertussis, DegP supports the two-partner secretion pathway of filamentous hemagglutinin (41). In enteropathogenic bacteria, including Helicobacter pylori, Campylobacter jejuni, and enteropathogenic E. coli, DegP is secreted and cleaves E-cadherin to disrupt intercellular adhesion, facilitating bacterial invasion (53). DegP mutants display defects in processing certain virulence factors, susceptibility to oxidative and thermal stresses, as well as compromised biofilm formation and adherence to epithelial cells. Consequently, many pathogenic bacteria deficient in functional DegP are either nonvirulent or less virulent (54).

In our study, we showed that the *in vivo* symbiotic function of *degP* was dependent on not the chaperone activity but the proteolytic activity of DegP. Using E. coli DegP, some authors claimed that DegP functions more as a protease, with hardly detectable chaperone activity (55, 56). They argued that the rescue of the lethal effects of assembly-defective outer membrane proteins by the expression of protease-deficient DegP^S210A^ (57, 58) was due to chaperone-like activity, sequestering defective outer membrane proteins, but not true chaperone activity, which promotes their normal assembly (55). Even if *Burkholderia* DegP did not have true chaperone activity, the chaperone-like activity of DegP would still function in sequestering and trafficking proteins to the outer membrane. However, the chaperone-like activity of DegP showed a negligible role in the *Burkholderia*-bean bug symbiosis. These results imply that the role of DegP in secreting proteins or expressing outer membrane proteins, which was important for several pathogens, may not be crucial for the *Burkholderia* symbiont. Rather, the proteolytic activity of DegP in stress resistance and biofilm formation is critical for the *Burkholderia* symbiont to persist in the host M4 midgut.

## MATERIALS AND METHODS

### Bacteria, plasmids, and media.

The bacterial strains and plasmids used in this study are listed in Table S1 in the supplemental material. Escherichia coli cells were cultured at 37°C in LB medium (1% [wt/vol] tryptone, 0.5% [wt/vol] yeast extract, and 0.5% [wt/vol] NaCl). *Burkholderia insecticola* cells were cultured at 30°C in YG medium (0.5% [wt/vol] yeast extract, 0.4% [wt/vol] glucose, and 0.1% [wt/vol] NaCl) unless indicated otherwise. The following antibiotics were used: 30 μg/mL of rifampicin, 50 μg/mL of kanamycin, and 30 μg/mL of chloramphenicol.

### Insect rearing.

Bean bugs were reared at 27°C under a long-day regimen of 16 h of light and 8 h of darkness and fed with soybean seeds and DWA (distilled water containing 0.05% ascorbic acid). Nymphal insects were reared in clear plastic containers (34 cm long, 19.5 cm wide, and 27.5 cm high). Upon reaching adulthood, the insects were transferred to larger containers (35 cm long, 35 cm wide, and 40 cm high), where cotton pads were attached to the walls for egg laying. Eggs were collected daily and transferred to new cages for hatching.

### *Burkholderia* symbiont inoculation.

*B. insecticola* strain RPE75 was cultured at 30°C in YG medium containing rifampicin. An inoculum solution was prepared by suspending mid-log-phase cultured *Burkholderia* cells in DWA at a concentration of 10^7^ cells/mL. Wet cotton balls soaked with the inoculum solution were provided to the thirsty newly molted second-instar nymphs. The thirst of the nymphs was generated by depriving DWA from rearing containers overnight. After 2 days of inoculation, the wet cotton balls were replaced with normal DWA. To generate aposymbiotic insects not harboring *Burkholderia* cells, we did not provide the *Burkholderia* inoculum solution but provided only sterile DWA.

### Isolation of symbiotic *Burkholderia* cells from the M4 midgut.

M4 midguts were dissected from 5th-instar nymphs and placed into 50 μL of 10 mM phosphate buffer (PB) (pH 7.0). M4 midguts were cut into pieces with fine scissors, and 1 mL of PB was added to the M4 pieces by gentle pipetting to release *Burkholderia* symbionts into the solution. The solution was then filtered through a 5-μm-pore-size filter to remove the host tissues, and *Burkholderia* cells were further washed with PB.

### Measurement of *degP* gene expression by quantitative PCR.

Symbiotic cells isolated from the M4 midgut, stationary-phase cultured cells, and log-phase cultured cells were prepared to have 2 × 10^8^ cells per sample. Bacterial samples were treated with RNAprotect bacterial reagent (Qiagen Inc., Valencia, CA, USA) and centrifuged to collect the cell pellet. The cell pellets were treated with 100 μL of nuclease-free water containing 400 μg/mL of lysozyme and 100 μg/mL of proteinase K for 10 min at room temperature. Next, bacterial RNA was extracted from these samples using preheated RiboEx (GeneAll Biotechnology, Seoul, South Korea) according to the manufacturer’s instructions. The extracted RNA was treated with RNase-free DNase I (Illumina, San Diego, CA, USA) for 15 min at 37°C and repurified with RiboEx. The cDNA was synthesized from the RNA samples using TOPscript RT DryMIX containing random hexamer primers (Enzynomics, Daejeon, South Korea). The cDNA templates were subjected to quantitative PCR (qPCR) using TOPreal qPCR 2× PreMIX with SYBR green (Enzynomics). The primer sequences (degP-qPCR-P1 and degP-qPCR-P2) used for qPCR are listed in Table S2. The PCR temperature profile was set to 95°C for 10 min followed by 40 cycles of 95°C for 10 s, 60°C for 15 s, and 72°C for 20 s using the CFX96 real-time PCR system (Bio-Rad, Hercules, CA, USA). The comparative threshold cycle (ΔΔ*C_T_*) method was used to analyze relative gene expression levels, and the *recA* gene (BRPE64_ACDS04710) of *B. insecticola* was used as an endogenous control gene.

### Generation of mutant strains.

The deletion of the chromosomal *degP* gene of *B. insecticola* was accomplished by homologous recombination followed by allelic exchange, utilizing the suicide vector pK18mobsacB, and complemented strains were made using the pBBR122 vector as described previously (22). Detailed methods for generating the deletion mutant and complemented mutant strains are described in the supplemental material.

### *In vivo* competition assay.

To test the competitiveness of the Δ*degP* mutant strain in symbiotic association against the wild-type stain, a mixed-inoculum solution (10^7^ cells/mL) with wild-type and Δ*degP* mutant cells (1:1 ratio) was prepared and provided to the 2nd-instar nymphs. At the 5th-instar nymphal stage, symbiotic *B. insecticola* cells were extracted from M4 midguts and spread onto a YG agar plate with rifampicin. Sixty colonies were randomly selected from each insect and examined for their *degP* deletion by PCR using primers degP-up and degP-down (Table S2).

### Measurement of bacterial growth in liquid media.

The growth curves of the *B. insecticola* strains were examined in either YG medium or M9 minimal medium (1.3% Na_2_HPO_4_·2H_2_O, 0.3% KH_2_PO_4_, 0.1% NH_4_Cl, 0.1% NaCl, 100 mM CaCl_2_, 100 mM MgSO_4_, 0.4% glucose) with the appropriate antibiotics. The starting cell solutions were prepared by adjusting the optical density at 600 nm (OD_600_) to 0.02 with stationary-phase cells and incubated at 27°C or 34°C for 47 h. The OD_600_ was monitored every hour using a Tecan Infinite M200 plate reader (Tecan Group Ltd., Männedorf, Switzerland).

### *In vitro* stress resistance assays.

For CFU assays, *B. insecticola* cells at mid-log phase grown in YG medium were washed with 10 mM PB (pH 7.0), the cell concentration was adjusted to 1 × 10^7^ cells/mL in PB, and the cells were treated for 18 h at room temperature under different stress conditions: 200 mM NaCl, 300 mM NaCl, or 500 mM sucrose. Cells incubated in PB for 18 h at room temperature were used as controls. After 18 h of treatment, cells were diluted and plated onto a YG agar plate, and CFU were counted. The survival rate was calculated as CFU_stress_/CFU_control_ × 100 (percent). The relative survival rates of the mutant strains were further calculated by comparing them to the survival rate of the wild-type strain: (survival rate)_mutant_/(survival rate)_WT_ × 100 (percent).

For stress assay using growth observation, the strains at mid-log phase grown in YG medium were washed with M9 medium, and the optical density at 600 nm was adjusted to 0.4. Ten microliters of the bacterial solution (OD_600_ of 0.4) was inoculated into 2 mL of M9 medium containing various concentrations of NaCl (12.5 mM, 25 mM, 50 mM, or 100 mM) and incubated for 2 days at 30°C with shaking. Bacterial samples cultured in M9 medium without NaCl were used as a control. After incubation, the optical density was measured at 600 nm. The survival rates with different concentrations of NaCl were calculated by comparing them with that of the control: (OD_600_)_salt treated_/(OD_600_)_control_ × 100 (percent).

### Microtiter plate biofilm assay.

A biofilm assay using crystal violet was performed as previously described, with slight modifications (16). Mid-log-phase *B. insecticola* strains were prepared by adjusting the OD_600_ to 0.8 in YG medium with the appropriate antibiotics, and 150 μL of the cell solution was added to each well of 96-well plates. The 96-well plates were incubated at 30°C for 48 h with shaking at 150 rpm. At the end of the incubation period, the OD_600_ values of each well were measured using a Tecan Infinite M200 plate reader. The 96-well plates were washed three times with PB, and the adherent biofilms were fixed with 99% methanol for 10 min. After removing the methanol and air drying, 200 μL of a 0.1% crystal violet solution was added to each well. The blank control was prepared by adding 150 μL of a crystal violet solution to the wells. After incubation for 20 min, the crystal violet solution was removed, and the wells were washed thoroughly with tap water and air dried. The biofilm-staining crystal violet was solubilized in 200 μL of 30% acetic acid, and the absorbance of each well solution was measured at 540 nm using a Tecan Infinite M200 plate reader.

### Generation, expression, and purification of recombinant proteins, DegP and protease-defective DegP^S248A^.

The *degP* gene carried on the pBBR122-degP complementation plasmid was amplified by PCR using primers listed in Table S2 and inserted into the pET28a expression vector, after being both cleaved with the NdeI and HindIII restriction enzymes and ligated to generate pET28a-degP. The N-terminal His tag sequence was added to *degP* from the plasmid. The E. coli DH5α cells transformed with pET28a-degP were selected on an LB agar plate with kanamycin. Site-directed mutagenesis to generate pET28a-degP^S248A^ was performed using primers degP-S248A-P1 and degP-S248A-P2 (Table S2).

The recombinant protein expression vectors pET28a-degP and pET28a-degP^S248A^ were transformed into an E. coli BL21(DE3)/pLysS strain (Table S1). To purify proteins, the transformed cells were grown at 37°C in LB medium containing 25 μg/mL kanamycin and 10 μg/mL chloramphenicol overnight and subcultured in LB medium without antibiotics for 2 to 3 h. At about mid-log phase (OD_600_ of 0.4), 0.4 mM isopropyl-β-d-thiogalactopyranoside was added, and the cells were further cultured for 3 h before being harvested by centrifugation.

The collected cells were resuspended in buffer A (50 mM NaH_2_PO_4_, 300 mM NaCl [pH 8.0]) and lysed by multiple freeze-thaw cycles. After being treated with DNase I, the lysed cells were centrifuged, and the supernatants were collected for affinity chromatography. A Ni-nitrilotriacetic acid (NTA) agarose column (Roche) was loaded with the supernatant, washed with buffer A, and eluted with buffer B (buffer A containing 250 mM imidazole). The eluates with good purity were selected by SDS-PAGE analysis and further dialyzed for buffer exchange. The protein concentration was determined using the bicinchoninic acid (BCA) protein assay kit (Pierce, Rockford, IL, USA).

### Protease assay.

The protease activities of DegP and DegP^S248A^ were measured by the degradation of β-casein (Sigma) as described previously, with some modifications (59). Two hundred microliters of a reaction solution containing 1.3 μM DegP or DegP^S248A^ and 13 μM β-casein in 50 mM HEPES (pH 7.8) was prepared and incubated at 37°C. At time points of 1, 30, 60, 120, and 240 min, 15 μL of the reaction solution was collected and mixed with 5 μL of SDS-PAGE sample buffer. β-Casein degradation by DegP was confirmed by SDS-PAGE analysis with a 13% polyacrylamide gel followed by staining with Coomassie brilliant blue.

### Light-scattering assay.

The chaperone activities of DegP and DegP^S248A^ were measured by a light-scattering assay using lysozyme and the denaturing reagent 1,4-dithiothreitol (DTT) as described previously, with slight modifications (60). A reaction solution containing 0.1 mg/mL of DegP or DegP^S248A^, 0.1 mg/mL of lysozyme, and 20 mM DTT in 50 mM Na_2_HPO_4_-NaH_2_PO_4_ (pH 7.6) was incubated at 37°C, and the reaction was monitored by measuring the absorbance at 360 nm with a Tecan Infinite M200 plate reader every 10 min for 1 h. For controls, reaction samples of lysozyme alone, lysozyme with DTT, DegP with DTT, and DegP^S248A^ with DTT were also monitored for protein aggregation by the OD_360_.

### CFU assay for estimating symbiont titers.

Individual M4 midguts dissected from bean bugs were collected in 100 μL of PB, homogenized using a microtube homogenizer, and serially diluted with PB. The diluted samples were spread onto YG agar plates with rifampicin. After 2 days of incubation at 30°C, the colonies on the plates were counted. The CFU per insect were calculated by multiplying the counted CFU by the dilution factor.

### Measurements of insect growth and fitness.

The adult emergence rate was monitored by inspecting late-5th-instar nymphs and counting the number of newly molted adult insects every day. On the second day after molting to an adult, the early-adult insects were anesthetized with CO_2_, and the insects were then immersed in acetone for 5 min and completely dried by incubating them in a 70°C oven, after which their dry body weights were measured.

### Statistical analyses.

Statistical analyses were performed using GraphPad Prism version 6.07 (GraphPad Software, San Diego, CA, USA). The significance of differences was assessed by one-way or two-way analysis of variance (ANOVA) with Tukey’s *post hoc* test for the comparison of multiple-group data. An unpaired *t* test was used to analyze the significance of differences between two groups. All experiments were repeated at least three times to confirm reproducibility.
