# Supplementary material for: Proteolytic Activity of DegP Is Required for the Burkholderia Symbiont To Persist in Its Host Bean Bug
Source: Microbiol Spectr. 2022 Dec 13;11(1):e04330-22. doi: 10.1128/spectrum.04330-22 (PMC9927360; doi:10.1128/spectrum.04330-22)

Supplementary Information

Proteolytic activity of DegP is required for the *Burkholderia* symbiont to persist in its host bean bug

Bohyun Jeong, Ho Am Jang, Junbeom Lee, Ha Ram Bae, Jiyeun Kate Kim

SUPPLEMENTARY METHODS

Generation of mutant strains. To generate the *degP* deletion mutant strain, the 5' and 3' regions of *degP* were first amplified from the *B. insecticola* RPE75 by PCR, using the primers listed in Table S2. After transforming *E. coli* DH5α cells with pK18mobsacB vector containing 5' and 3' regions of *degP*, triparental conjugation was performed to transfer the cloned vector of donor *E. coli* DH5α cells to recipient RPE75 cells with conjugal help from helper HBL1 cells (Table S1). *Burkholderia* RPE75 cells with the first crossover were selected on the YG agar plates containing rifampicin and kanamycin. The second crossover was allowed by culturing the single crossover cells in the YG medium. Cells with the second crossover were selected on YG agar plates containing rifampicin and sucrose (200 μg/ml). The Δ*degP* deletion mutants by double crossover were identified by PCR.

To complement the Δ*degP* strain, we used the broad host range vector pBBR122 to clone the *degP* gene (Table S1). PCR insert of *degP* was prepared using the primers for complementation (degP-com-P1 and degP-com-P2, Table S2). The amplified DNA fragments were cloned into pBBR122, and the cloned vector was transformed into *E. coli* DH5α cells. To generate pBBR122-degP^{S248A} complement plasmid, pBBR122-degP plasmid was subjected to site-directed mutagenesis using primers, degP-S248A-P1 and degP-S248A-P2, listed in Table S2. Using a triparental conjugation, pBBR122-degP or pBBR122-degP^{S248A} complement plasmids were transferred to the recipient RPE75 Δ*degP* strain. The complemented cells were selected on YG agar plates containing rifampicin and kanamycin.

Disk diffusion assay. Susceptibility of *B. insecticola* to surfactant and oxidative stress was measured by disk diffusion assay. *B. insecticola* cells were cultured to the end of log phase (OD_{600}, 0.1). One milliliter of cultured cell solution was mixed with 50 ml of unsolidified YG-agar media to achieve a final OD_{600} of 0.02. Three milliliters of cell-YG-agar solution was poured into 35 mm dishes and allowed to solidify. For the surfactant assay, different concentrations of sodium dodecyl sulfate (SDS) were applied to disks placed in the middle of the dishes. For the oxidative stress assay, different concentrations of hydrogen peroxide (H_2O_2) were applied to the disks. After incubating dishes at 30°C for 24 h, the diameters of inhibition zones were measured.

Motility assay. Five microliters of mid-log *B. insecticola* cells (OD_{600}, 0.3) were injected into the middle of soft YG-agar plate prepared with 0.2% agar. Plates were cultured for 48 h, and the diameters of swimming zones were measured.

Table S1. Bacterial strains and plasmids used in this study.

Bacterial strains or plasmids	Characteristics	Reference[a]
Burkholderia insecticola		
RPE75	*B. insecticola* (RPE64); Rif^R	[1]
BKJ001	RPE75 *ΔdegP*; Rif^R	This study
Escherichia coli		
DH5α	F–Φ80*lac*ZΔM15 Δ(*lac*ZYA-*arg*F) U169 *rec*A1 *end*A1 *hsd*R17 (rK–, mK+) *pho*A *sup*E44 λ– *thi*-1 *gyr*A96 *rel*A1	Invitrogen
PIR1	F- *Δlac*169 *rpo*S(am) *rob*A1 *cre*C510 *hsd*R514 *end*A *rec*A1 *uidA(ΔMlu I)::pir-116*	Invitrogen
HBL1	PIR1 carrying pSTV28 and pEVS104; Cm^R, Km^R	[2]
BL21(DE3) pLysS	F$^-$ *omp*T *hsd*SB (r_B^-, m_B^-) *galdcmrne*131 (DE3) pLysS (Cam^R)	Invitrogen
Plasmids		
pSTV28	p15Aori; Cm^R	Takara
pEVS104	oriR6K helper plasmid containing conjugal *tra* and *trb*; Km^R	[3]
pK18mobsacB	pMB1ori allelic exchange vector containing oriT; Km^R	[4]
pBBR122	Broad host range vector; Cm^R, Km^R	[5]
pET28a	His_6-tagged protein expression vector; Km^R	Novagen

[a] [1] Kikuchi et al. (2011) ISME J 5, 446-460
[2] Kim et al. (2013) PNAS 110, E2381-2389
[3] Stabb and Ruby (2002) Methods Enzymol 358, 413-426
[4] Schäfer et al. (1994) J Bacteriol 176, 7309-7319
[5] Szpirer et al. (2001) J Bacteriol 183, 2101-2110

Table S2. PCR primers used in this study.

PCR target region or purpose	Primer name	Sequence (5'-3')
degP	degP-qPCR-P1	ACCGCTTTTCAATCTGCAAG
	degP-qPCR-P2	TCACCTTGATCGCTTCATTG
recA	recA-qPCR-P1	GTCGAAGACATCCAGGTGGT
	recA-qPCR-P2	ACTCCGGACCGTAGATTTCC
5' region of degP	degP-L-P1	CCCGGATCCCTCGATCTCGAAGGACATCA
	degP-L-P2	CCCTCTAGAACATGCTTCGCGCTGATATT
3' region of degP	degP-R-P1	CCCTCTAGACAATGAAGCGATCAAGGTGA
	degP-R-P2	CCCAAGCTTTCTGCTCCTTTTTCCGATGT
degP complementation	degP-com-P1	GCCGGAAGCGAAGAAGTAGA
	degP-com-P2	GTCGACTTCACGCGATAGG
degP recombinant protein	degP-recombinant-P1	GCCATATGGGCCATAACAATGTTCCTGCG
	degP-recombinant-P2	GCAAGCTTTCAGCTCAGATCGACCGGCAC
degP S248A point mutation	degP-S248A-P1	GTGAATCCGGGCAACGCGGGCGGACCGCTTTT
	degP-S248A-P2	AAAAGCGGTCCGCCCGCGTTGCCCGGATTCAC
degP deletion check	degP-up	ACCGACGATCCTCGAAAAG
	degP-down	GACGTATCGTTCGTGGTTCC

Figure S1. Surfactant and oxidative stress assays of wild-type and Δ*degP* strains of *B. insecticola*. Bacterial growth inhibition by surfactant (SDS) (**A**) and oxidative stress (H_2O_2) (**B**) is indicated by clear halos. The similar diameters of clear halos between wild-type and Δ*degP* strains indicate no difference in susceptibility to SDS or H_2O_2.

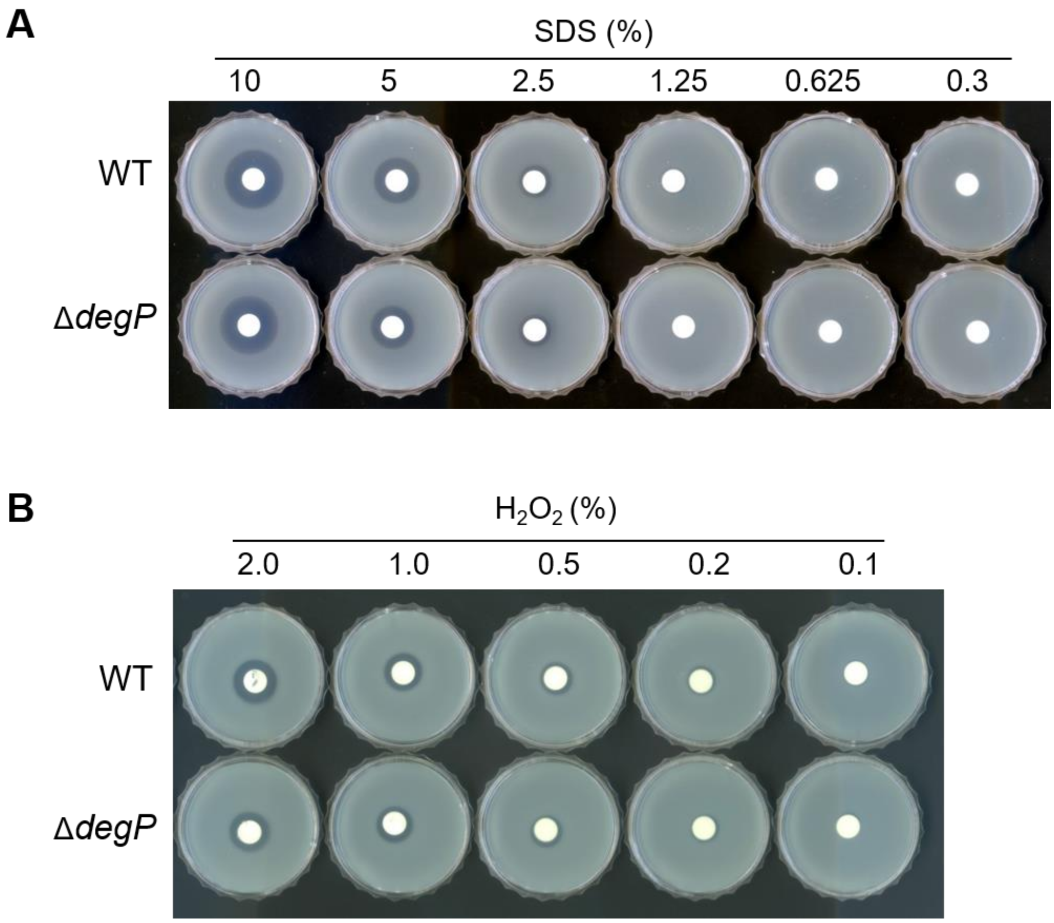

Figure S2. Motility assay of wild-type and Δ*degP* strains of *B. insecticola*. Bacterial motilities are determined by measuring diameters of opaque halos. There were no statistical differences in swimming diameters between the two strains in 1-day and 2-day incubation (unpaired t test; NS, not significant). Means and SDs (n = 7) are shown as columns and error bars, respectively.

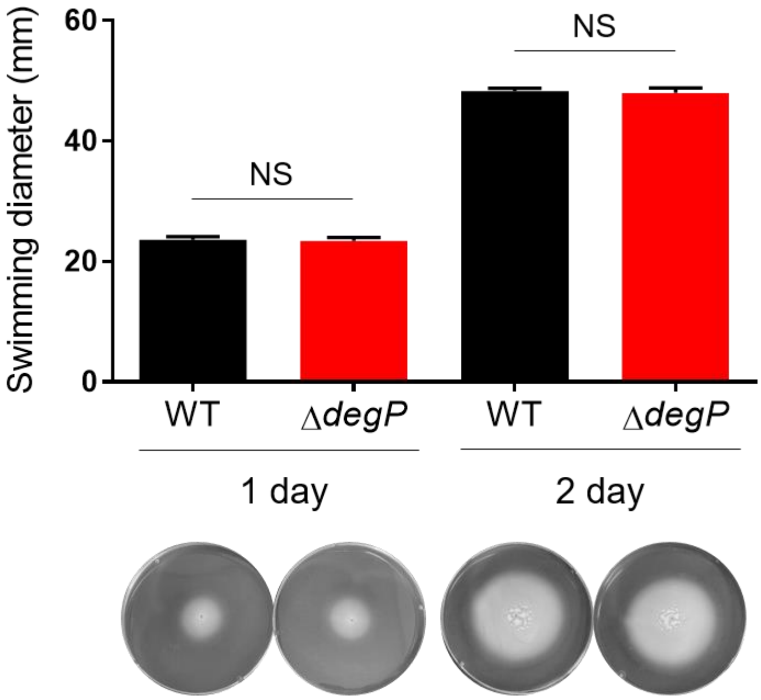

Supplement: Supplemental file 1 — Supplemental material. Download spectrum.04330-22-s0001.pdf, PDF file, 0.5 MB [file spectrum.04330-22-s0001.pdf]
